# Supplementary material for: Computational modeling of cambium activity provides a regulatory framework for simulating radial plant growth
Source: eLife. 2023 Mar 10;12:e66627. doi: 10.7554/eLife.66627 (PMC10069871; doi:10.7554/eLife.66627)
Supplement: Supplementary file 2. [file elife-66627-supp2.docx]

**Parameter values and chemical thresholds after parameter estimation (Model 4).**

| Parameter | description | Set1 | Set2 | Set3 | Set4 | Set5 |
| --- | --- | --- | --- | --- | --- | --- |
| k0 | chemical 6 limit above which parenchyma cells can divide | 0.000171 | 7.67E-05 | 0.000109 | 8.78E-05 | 2.07E-04 |
| k1 | xylem maximal cell size | 1.82796 | 1.86595 | 2.95035 | 1.76457 | 1.27415 |
| k2 | cell growth rate | 5 | 5 | 5 | 5 | 5 |
| k3 | used to scan the different cell wall stability values of the xylem stiffness regime | NA | NA | NA | NA | NA |
| k4 | chemical 5 limit below which parenchyma can convert to phloem poles | 7.64E-07 | 1.14E-06 | 2.45E-06 | 2.38E-06 | 2.62E-06 |
| k5 | chemical 6 limit above which cambium cells can turn into parenchyma | 0.036408 | 0.024388 | 0.053574 | 0.068591 | 0.046244 |
| k6 | chemical 6 limit above which cambium cells can divide | 3.34E-05 | 9.46E-05 | 2.03E-04 | 1.37E-04 | 2.60E-04 |
| k7 | phloem parenchyma maximal cell size | 4.84342 | 6.90276 | 7.64855 | 3.1102 | 1.53439 |
| k8 | size above which parenchyma is converted to phloem poles | 1.09831 | 2.08309 | 0.740948 | 0.885084 | 1.04212 |
| k9 | phloem pole maximal cell size | 6.48873 | 3.52371 | 6.12043 | 1.58445 | 6.35127 |
| k10 | cambium maximal cell size | 4.21208 | 4.25555 | 2.52557 | 5.8849 | 4.43106 |
| k11 | cambium cell size limit above which it can convert to xylem | 1.37943 | 1.11513 | 1.14325 | 0.732934 | 1.34715 |
| k12 | chemical 1 limit above which cambium cell can convert into xylem | 3.38481 | 8.37199 | 6.90236 | 4.1028 | 4.61511 |
| k13 | inhibition constant of how much chemical 4 suppresses PXY expression | 104.181 | 118.07 | 67.3786 | 113.375 | 84.444 |
| k14 | rate of how much PXY stimulates the production of DF (*pxy* mutant = 0) | 96.0397 | 136.717 | 181.138 | 222.365 | 86.9136 |
| k15 | cell size above which parenchyma can divide | 0.550294 | 0.601247 | 0.831244 | 0.645843 | 0.506946 |
| k16 | cell size above which cambium cells can divide | 0.795801 | 1.08617 | 1.08633 | 0.637028 | 0.860049 |
| k17 | defines the saturation curve for chemical 6 | 0.038657 | 0.04256 | 0.024591 | 0.036093 | 0.009327 |
| k18 | defines the saturation curve for chemical 6 | 20.3659 | 38.1824 | 23.8465 | 21.9007 | 25.1881 |
| k19 | CLE41 production rate in phloem parenchyma | 0.210723 | 0.163262 | 0.286353 | 0.299001 | 0.187163 |
| k20 | CLE41production rate in phloem poles | 1.59264 | 2.7594 | 0.875289 | 1.05448 | 0.718633 |
| K21 | diffusion rate of CLE41 | 0.000217 | 0.000104 | 0.000143 | 9.87E-05 | 0.000352 |
| K22 | degradation rate of CLE41 | 0.166462 | 0.124361 | 0.198326 | 0.116398 | 0.163 |
